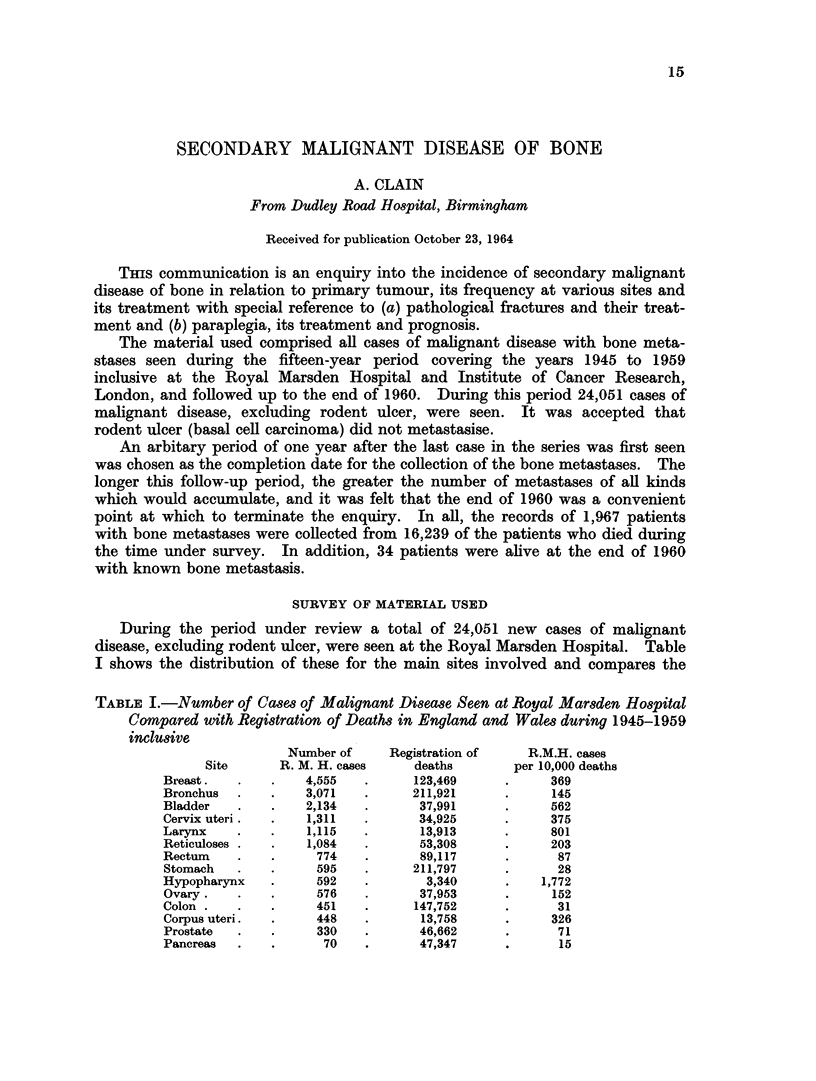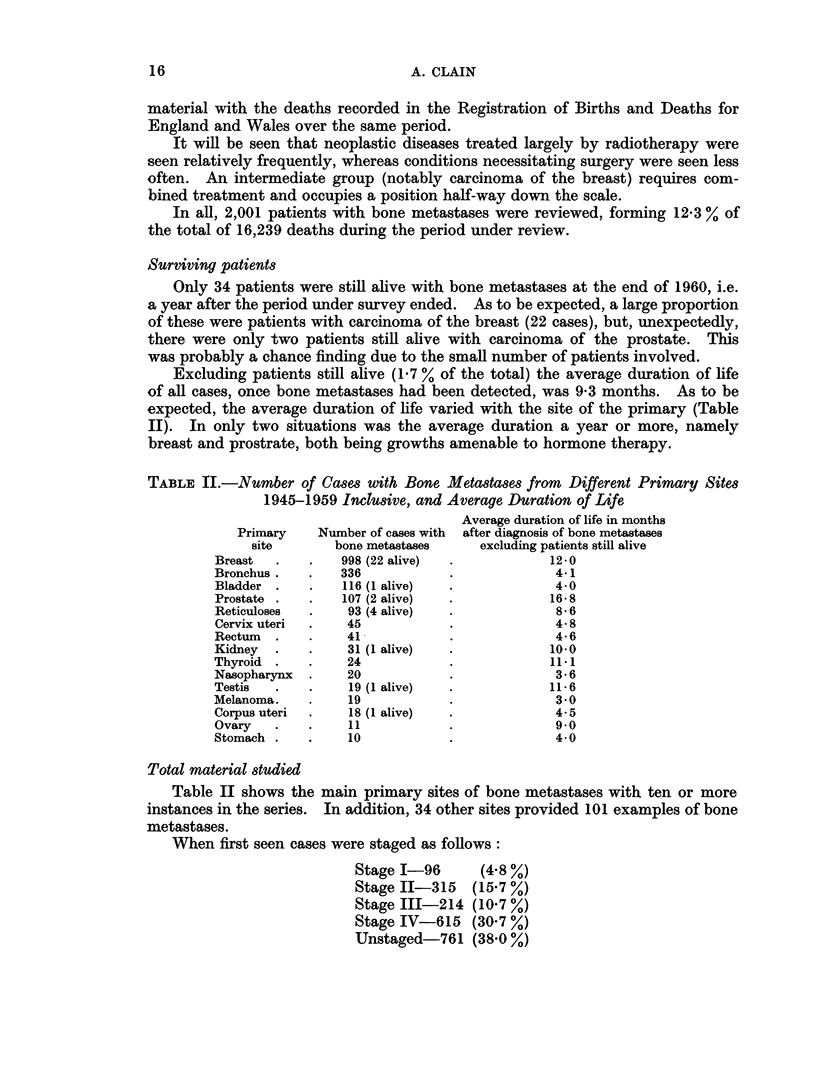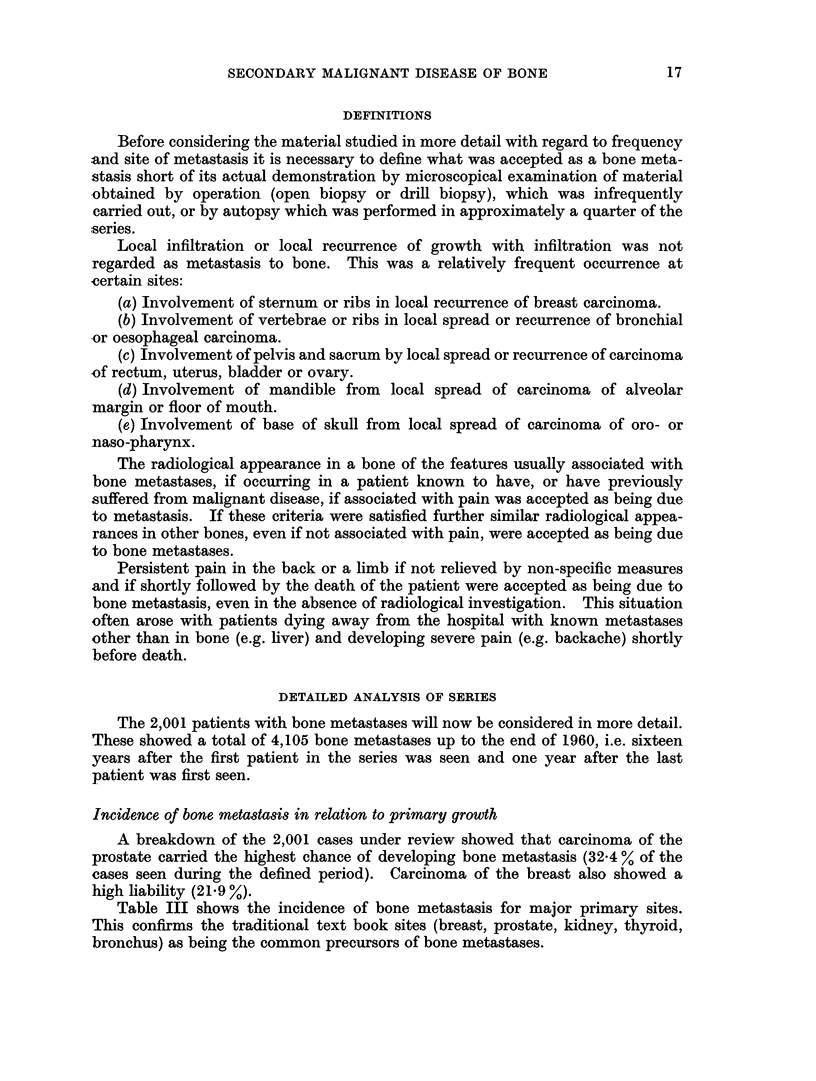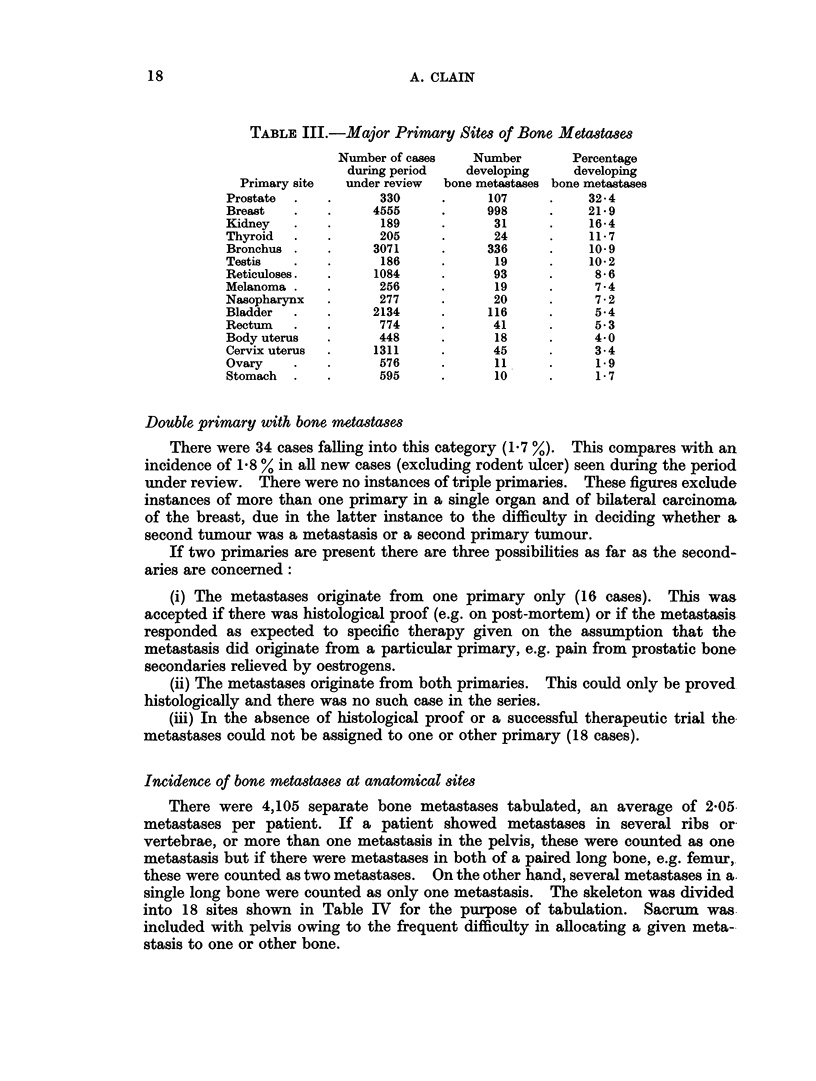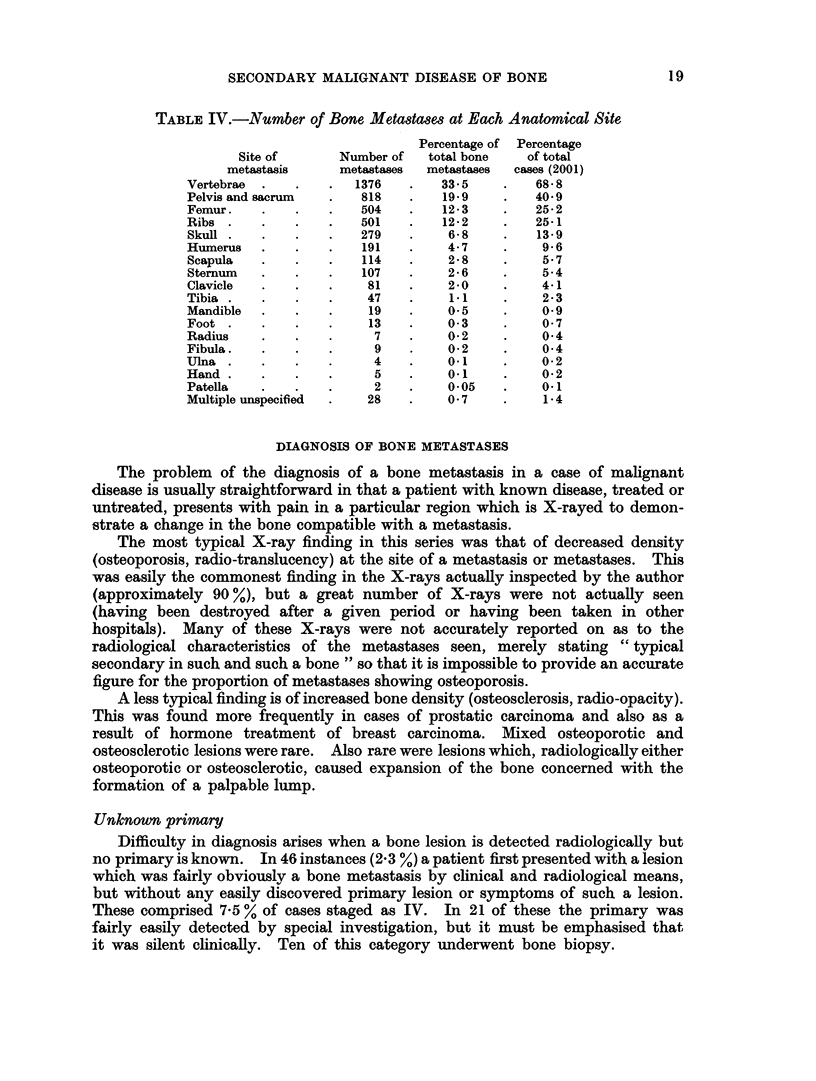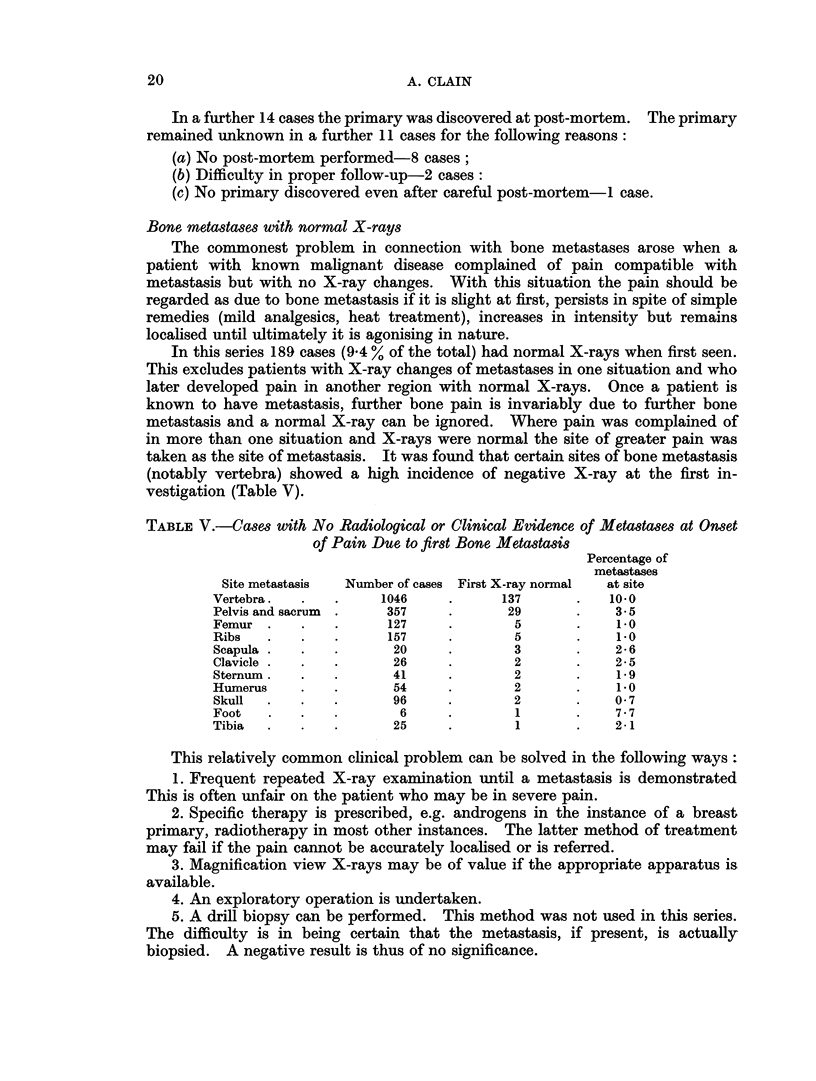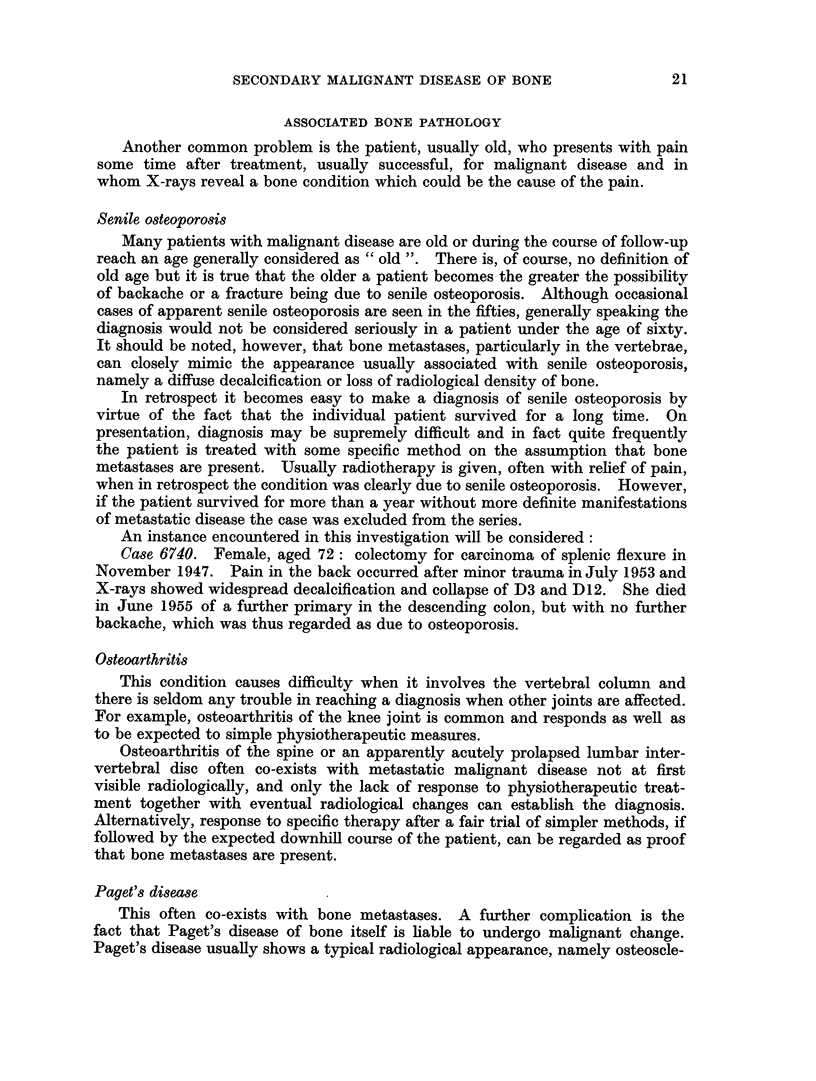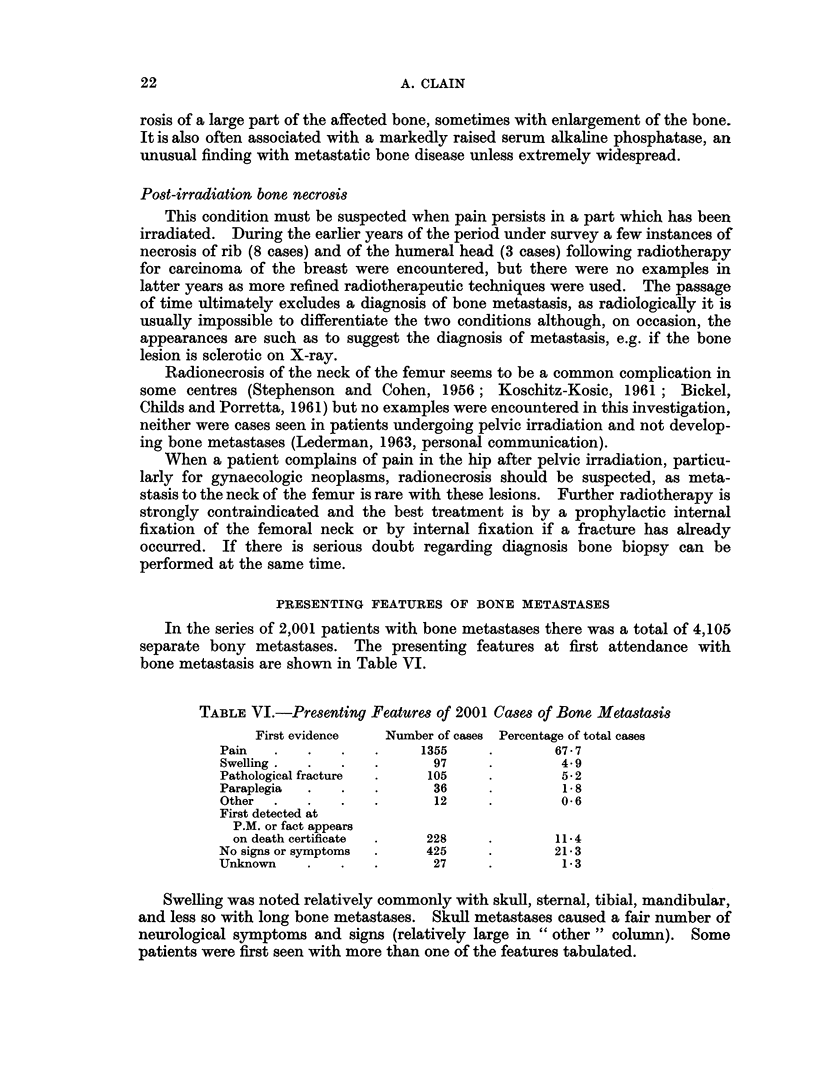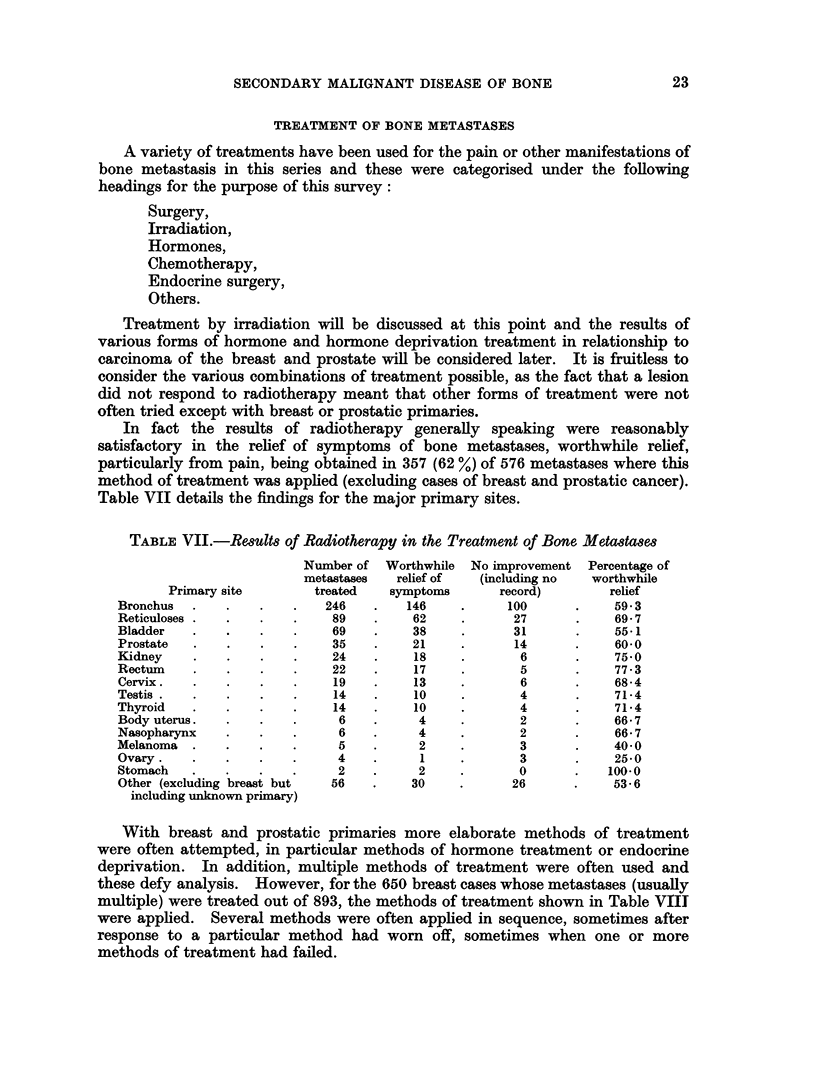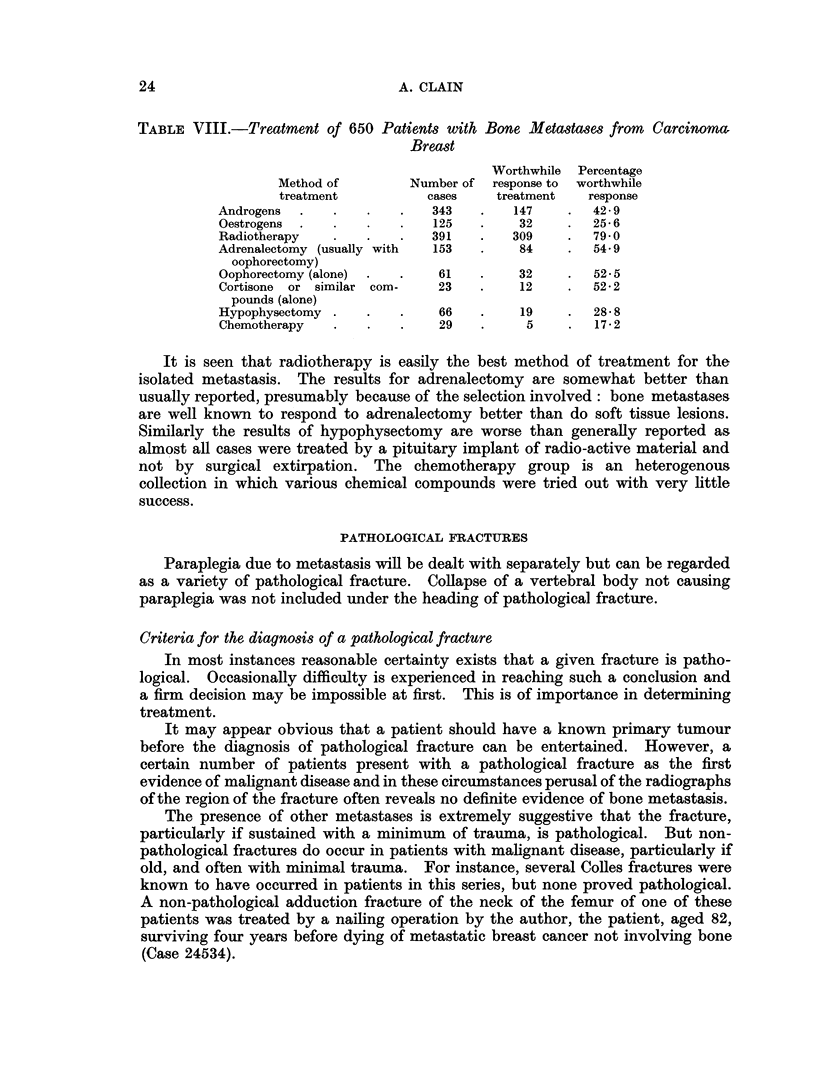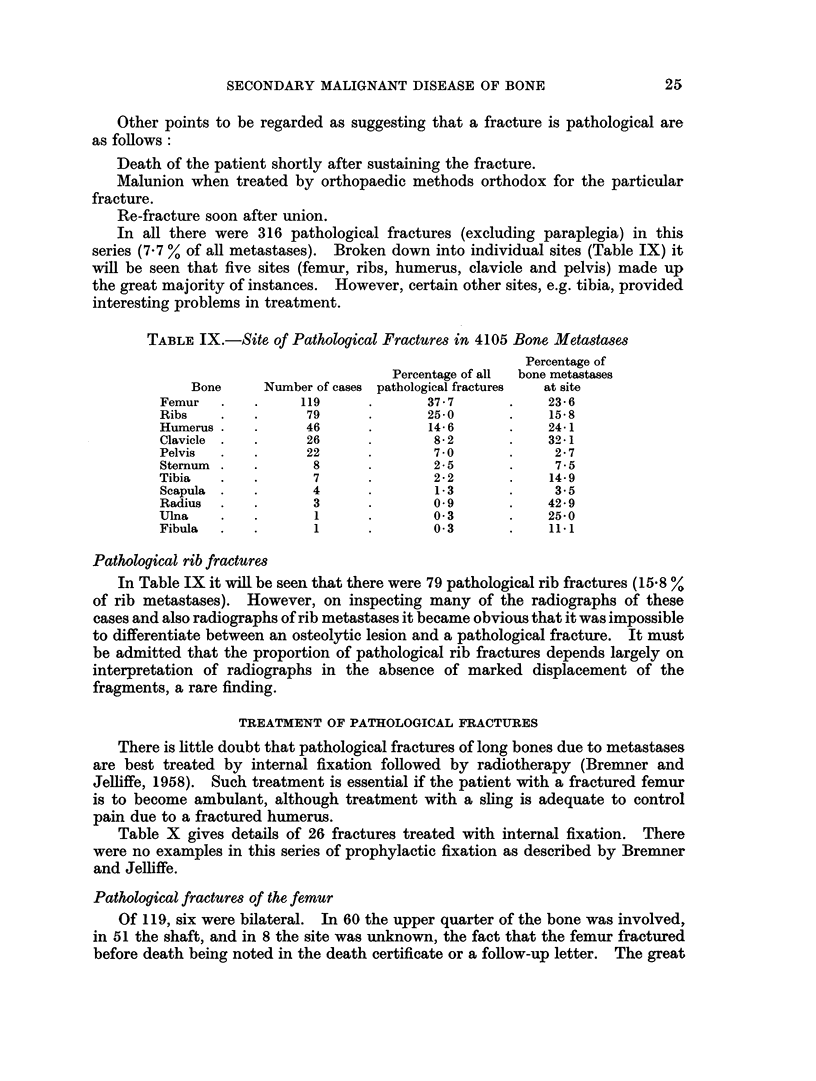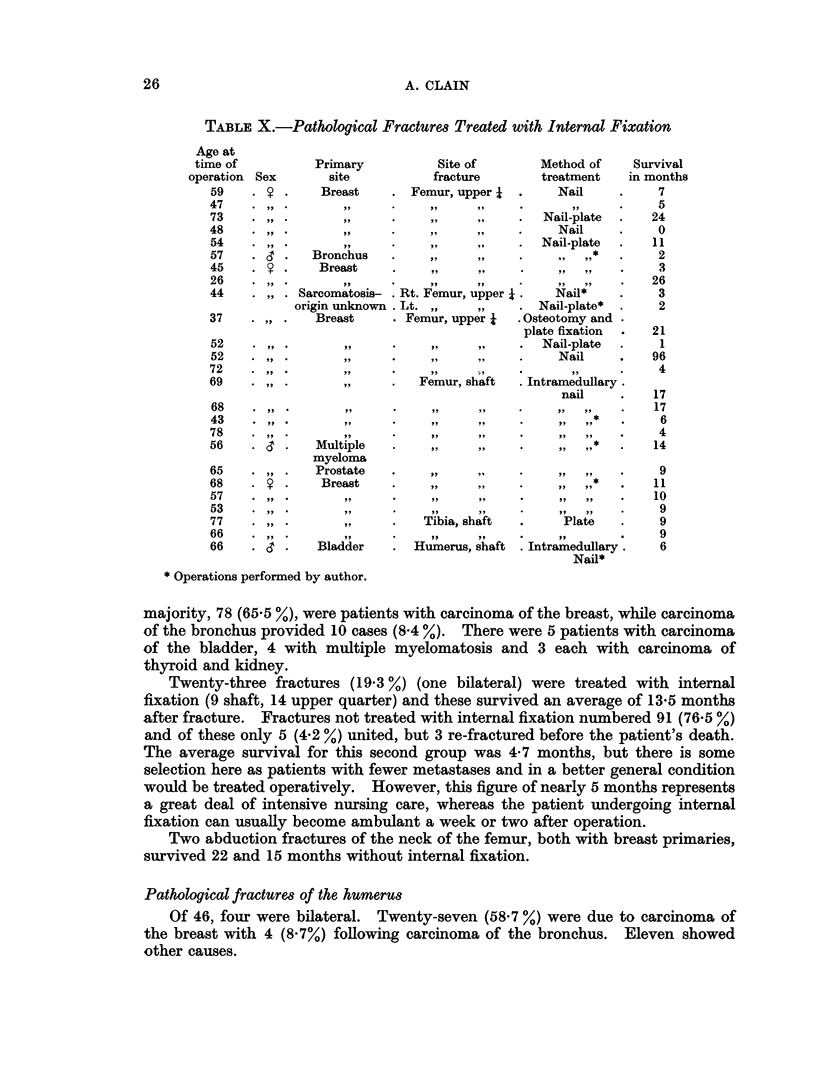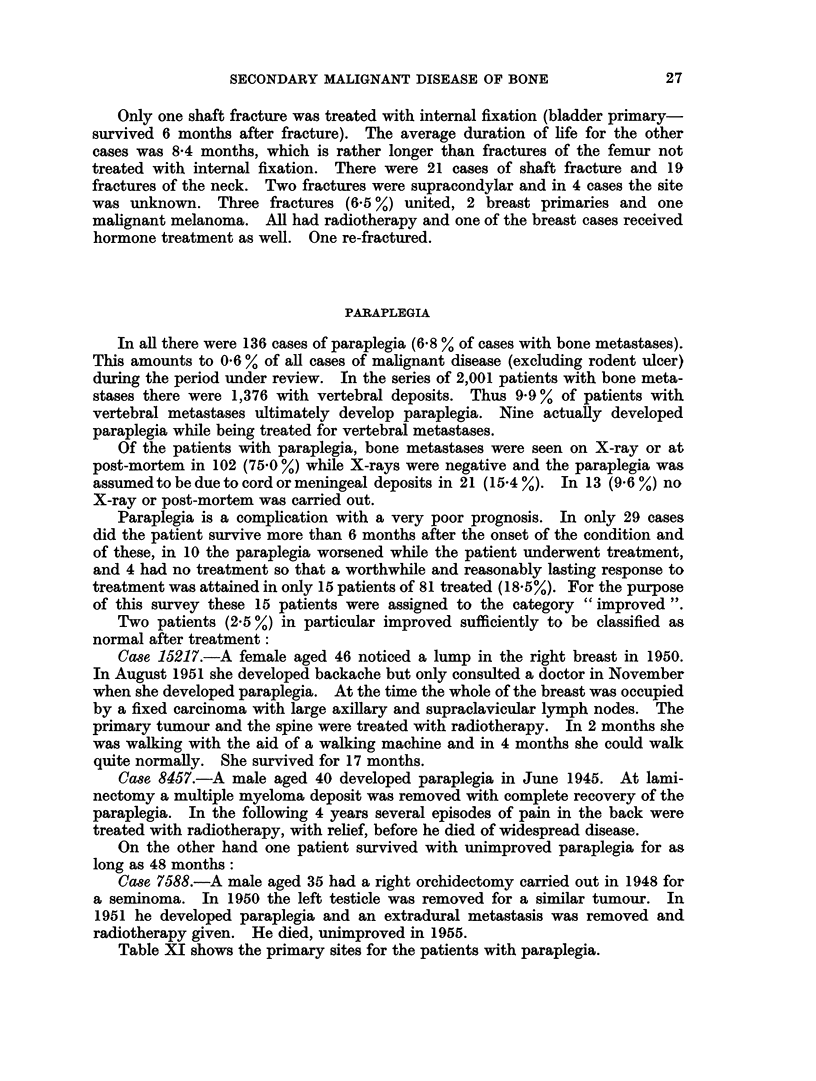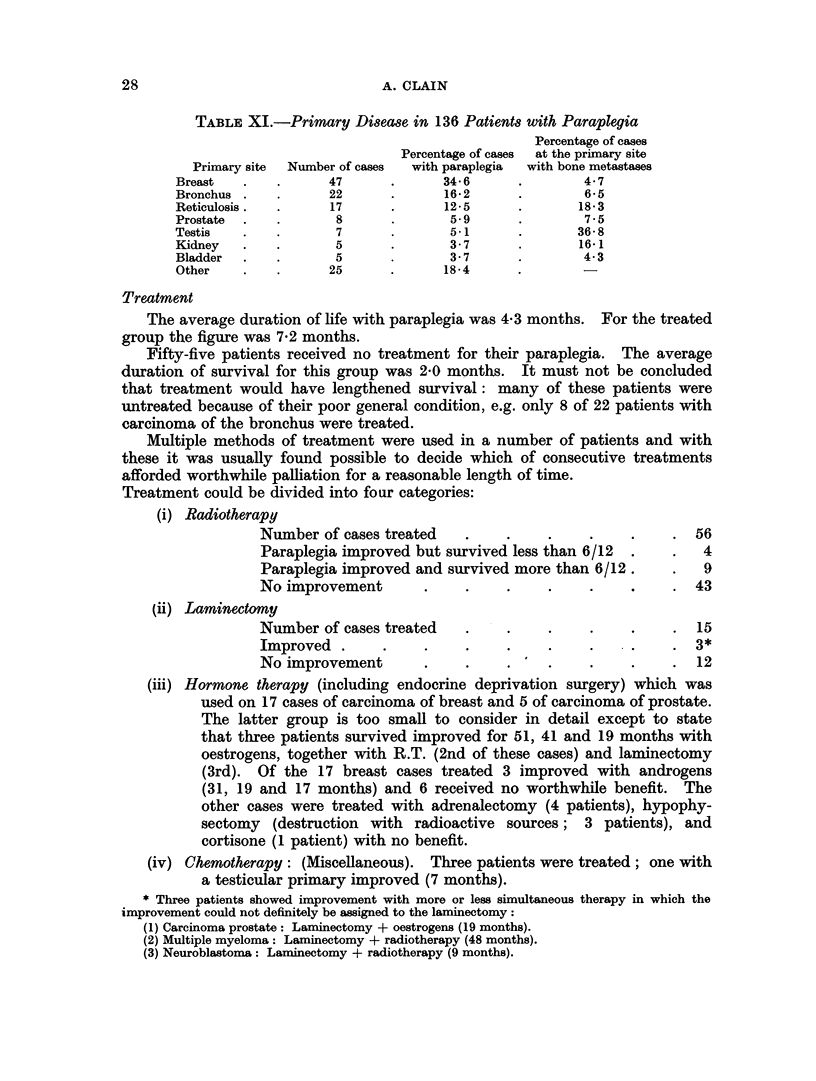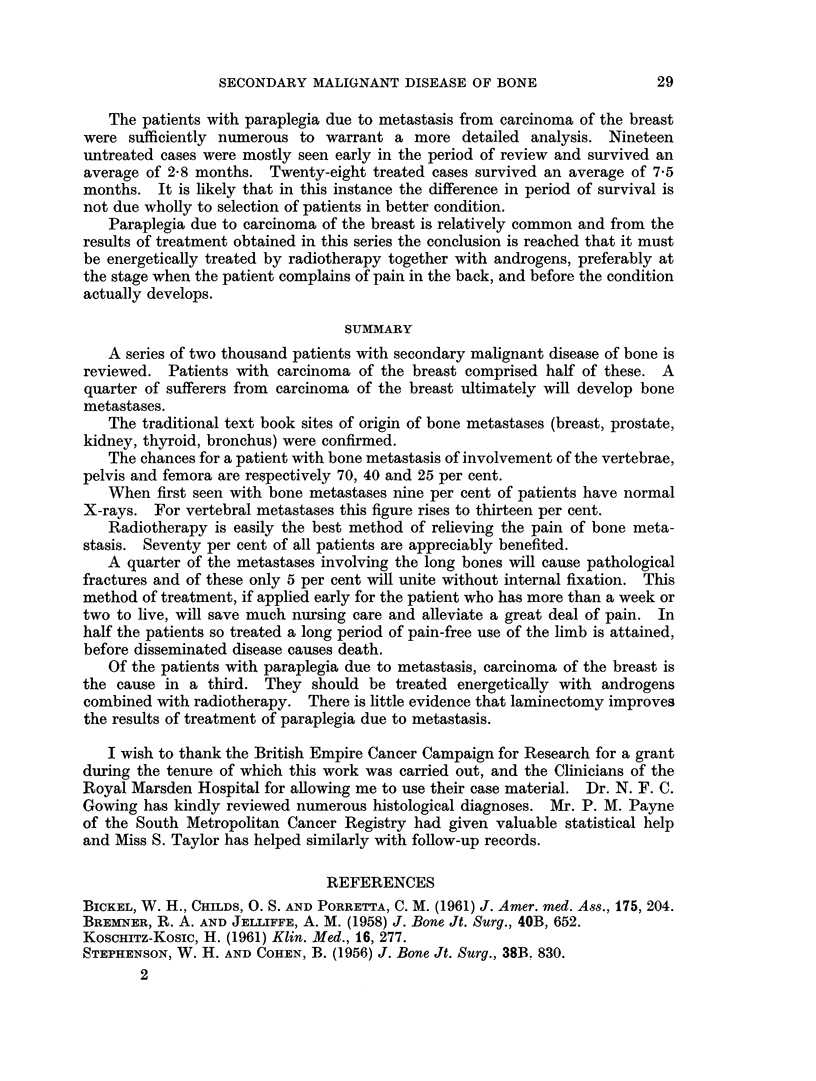# Secondary Malignant Disease of Bone

**DOI:** 10.1038/bjc.1965.3

**Published:** 1965-03

**Authors:** A. Clain


### SECONDARY MALIGNANT DISEASE OF BONE

A. CLAIN

*From Dudley Road Hospital, Birmingham*

Received for publication October 23, 1964

THIS communication is an enquiry into the incidence of secondary malignant disease of bone in relation to primary tumour, its frequency at various sites and its treatment with special reference to (*a*) pathological fractures and their treatment and (*b*) paraplegia, its treatment and prognosis.

The material used comprised all cases of malignant disease with bone metastases seen during the fifteen-year period covering the years 1945 to 1959 inclusive at the Royal Marsden Hospital and Institute of Cancer Research, London, and followed up to the end of 1960. During this period 24,051 cases of malignant disease, excluding rodent ulcer, were seen. It was accepted that rodent ulcer (basal cell carcinoma) did not metastasise.

An arbitary period of one year after the last case in the series was first seen was chosen as the completion date for the collection of the bone metastases. The longer this follow-up period, the greater the number of metastases of all kinds which would accumulate, and it was felt that the end of 1960 was a convenient point at which to terminate the enquiry. In all, the records of 1,967 patients with bone metastases were collected from 16,239 of the patients who died during the time under survey. In addition, 34 patients were alive at the end of 1960 with known bone metastasis.

### SURVEY OF MATERIAL USED

During the period under review a total of 24,051 new cases of malignant disease, excluding rodent ulcer, were seen at the Royal Marsden Hospital. Table I shows the distribution of these for the main sites involved and compares the

TABLE I.—*Number of Cases of Malignant Disease Seen at Royal Marsden Hospital Compared with Registration of Deaths in England and Wales during* 1945–1959 *inclusive*

| Site | Number of R. M. H. cases | Registration of deaths | R.M.H. cases per 10,000 deaths |
|---|---|---|---|
| Breast | 4,555 | 123,469 | 369 |
| Bronchus | 3,071 | 211,921 | 145 |
| Bladder | 2,134 | 37,991 | 562 |
| Cervix uteri | 1,311 | 34,925 | 375 |
| Larynx | 1,115 | 13,913 | 801 |
| Reticuloses | 1,084 | 53,308 | 203 |
| Rectum | 774 | 89,117 | 87 |
| Stomach | 595 | 211,797 | 28 |
| Hypopharynx | 592 | 3,340 | 1,772 |
| Ovary | 576 | 37,953 | 152 |
| Colon | 451 | 147,752 | 31 |
| Corpus uteri | 448 | 13,758 | 326 |
| Prostate | 330 | 46,662 | 71 |
| Pancreas | 70 | 47,347 | 15 |

material with the deaths recorded in the Registration of Births and Deaths for England and Wales over the same period.

It will be seen that neoplastic diseases treated largely by radiotherapy were seen relatively frequently, whereas conditions necessitating surgery were seen less often. An intermediate group (notably carcinoma of the breast) requires combined treatment and occupies a position half-way down the scale.

In all, 2,001 patients with bone metastases were reviewed, forming 12·3 % of the total of 16,239 deaths during the period under review.

*Surviving patients*

Only 34 patients were still alive with bone metastases at the end of 1960, i.e. a year after the period under survey ended. As to be expected, a large proportion of these were patients with carcinoma of the breast (22 cases), but, unexpectedly, there were only two patients still alive with carcinoma of the prostate. This was probably a chance finding due to the small number of patients involved.

Excluding patients still alive (1·7 % of the total) the average duration of life of all cases, once bone metastases had been detected, was 9·3 months. As to be expected, the average duration of life varied with the site of the primary (Table II). In only two situations was the average duration a year or more, namely breast and prostrate, both being growths amenable to hormone therapy.

TABLE II.—*Number of Cases with Bone Metastases from Different Primary Sites 1945–1959 Inclusive, and Average Duration of Life*

| Primary site | Number of cases with bone metastases | Average duration of life in months after diagnosis of bone metastases excluding patients still alive |
|---|---|---|
| Breast | 998 (22 alive) | 12·0 |
| Bronchus | 336 | 4·1 |
| Bladder | 116 (1 alive) | 4·0 |
| Prostate | 107 (2 alive) | 16·8 |
| Reticuloses | 93 (4 alive) | 8·6 |
| Cervix uteri | 45 | 4·8 |
| Rectum | 41 | 4·6 |
| Kidney | 31 (1 alive) | 10·0 |
| Thyroid | 24 | 11·1 |
| Nasopharynx | 20 | 3·6 |
| Testis | 19 (1 alive) | 11·6 |
| Melanoma | 19 | 3·0 |
| Corpus uteri | 18 (1 alive) | 4·5 |
| Ovary | 11 | 9·0 |
| Stomach | 10 | 4·0 |

*Total material studied*

Table II shows the main primary sites of bone metastases with ten or more instances in the series. In addition, 34 other sites provided 101 examples of bone metastases.

When first seen cases were staged as follows :

Stage I—96 (4·8 %)
Stage II—315 (15·7 %)
Stage III—214 (10·7 %)
Stage IV—615 (30·7 %)
Unstaged—761 (38·0 %)

### DEFINITIONS

Before considering the material studied in more detail with regard to frequency and site of metastasis it is necessary to define what was accepted as a bone metastasis short of its actual demonstration by microscopical examination of material obtained by operation (open biopsy or drill biopsy), which was infrequently carried out, or by autopsy which was performed in approximately a quarter of the series.

Local infiltration or local recurrence of growth with infiltration was not regarded as metastasis to bone. This was a relatively frequent occurrence at certain sites:

(*a*) Involvement of sternum or ribs in local recurrence of breast carcinoma.

(*b*) Involvement of vertebrae or ribs in local spread or recurrence of bronchial or oesophageal carcinoma.

(*c*) Involvement of pelvis and sacrum by local spread or recurrence of carcinoma of rectum, uterus, bladder or ovary.

(*d*) Involvement of mandible from local spread of carcinoma of alveolar margin or floor of mouth.

(*e*) Involvement of base of skull from local spread of carcinoma of oro- or naso-pharynx.

The radiological appearance in a bone of the features usually associated with bone metastases, if occurring in a patient known to have, or have previously suffered from malignant disease, if associated with pain was accepted as being due to metastasis. If these criteria were satisfied further similar radiological appearances in other bones, even if not associated with pain, were accepted as being due to bone metastases.

Persistent pain in the back or a limb if not relieved by non-specific measures and if shortly followed by the death of the patient were accepted as being due to bone metastasis, even in the absence of radiological investigation. This situation often arose with patients dying away from the hospital with known metastases other than in bone (e.g. liver) and developing severe pain (e.g. backache) shortly before death.

### DETAILED ANALYSIS OF SERIES

The 2,001 patients with bone metastases will now be considered in more detail. These showed a total of 4,105 bone metastases up to the end of 1960, i.e. sixteen years after the first patient in the series was seen and one year after the last patient was first seen.

#### *Incidence of bone metastasis in relation to primary growth*

A breakdown of the 2,001 cases under review showed that carcinoma of the prostate carried the highest chance of developing bone metastasis (32·4 % of the cases seen during the defined period). Carcinoma of the breast also showed a high liability (21·9 %).

Table III shows the incidence of bone metastasis for major primary sites. This confirms the traditional text book sites (breast, prostate, kidney, thyroid, bronchus) as being the common precursors of bone metastases.

TABLE III.—*Major Primary Sites of Bone Metastases*

| Primary site | Number of cases during period under review | Number developing bone metastases | Percentage developing bone metastases |
|---|---|---|---|
| Prostate | 330 | 107 | 32·4 |
| Breast | 4555 | 998 | 21·9 |
| Kidney | 189 | 31 | 16·4 |
| Thyroid | 205 | 24 | 11·7 |
| Bronchus | 3071 | 336 | 10·9 |
| Testis | 186 | 19 | 10·2 |
| Reticuloses | 1084 | 93 | 8·6 |
| Melanoma | 256 | 19 | 7·4 |
| Nasopharynx | 277 | 20 | 7·2 |
| Bladder | 2134 | 116 | 5·4 |
| Rectum | 774 | 41 | 5·3 |
| Body uterus | 448 | 18 | 4·0 |
| Cervix uterus | 1311 | 45 | 3·4 |
| Ovary | 576 | 11 | 1·9 |
| Stomach | 595 | 10 | 1·7 |

*Double primary with bone metastases*

There were 34 cases falling into this category (1·7 %). This compares with an incidence of 1·8 % in all new cases (excluding rodent ulcer) seen during the period under review. There were no instances of triple primaries. These figures exclude instances of more than one primary in a single organ and of bilateral carcinoma of the breast, due in the latter instance to the difficulty in deciding whether a second tumour was a metastasis or a second primary tumour.

If two primaries are present there are three possibilities as far as the secondaries are concerned :

(i) The metastases originate from one primary only (16 cases). This was accepted if there was histological proof (e.g. on post-mortem) or if the metastasis responded as expected to specific therapy given on the assumption that the metastasis did originate from a particular primary, e.g. pain from prostatic bone secondaries relieved by oestrogens.

(ii) The metastases originate from both primaries. This could only be proved histologically and there was no such case in the series.

(iii) In the absence of histological proof or a successful therapeutic trial the metastases could not be assigned to one or other primary (18 cases).

*Incidence of bone metastases at anatomical sites*

There were 4,105 separate bone metastases tabulated, an average of 2·05 metastases per patient. If a patient showed metastases in several ribs or vertebrae, or more than one metastasis in the pelvis, these were counted as one metastasis but if there were metastases in both of a paired long bone, e.g. femur, these were counted as two metastases. On the other hand, several metastases in a single long bone were counted as only one metastasis. The skeleton was divided into 18 sites shown in Table IV for the purpose of tabulation. Sacrum was included with pelvis owing to the frequent difficulty in allocating a given metastasis to one or other bone.

TABLE IV.—*Number of Bone Metastases at Each Anatomical Site*

| Site of metastasis | Number of metastases | Percentage of total bone metastases | Percentage of total cases (2001) |
|---|---|---|---|
| Vertebrae | 1376 | 33·5 | 68·8 |
| Pelvis and sacrum | 818 | 19·9 | 40·9 |
| Femur | 504 | 12·3 | 25·2 |
| Ribs | 501 | 12·2 | 25·1 |
| Skull | 279 | 6·8 | 13·9 |
| Humerus | 191 | 4·7 | 9·6 |
| Scapula | 114 | 2·8 | 5·7 |
| Sternum | 107 | 2·6 | 5·4 |
| Clavicle | 81 | 2·0 | 4·1 |
| Tibia | 47 | 1·1 | 2·3 |
| Mandible | 19 | 0·5 | 0·9 |
| Foot | 13 | 0·3 | 0·7 |
| Radius | 7 | 0·2 | 0·4 |
| Fibula | 9 | 0·2 | 0·4 |
| Ulna | 4 | 0·1 | 0·2 |
| Hand | 5 | 0·1 | 0·2 |
| Patella | 2 | 0·05 | 0·1 |
| Multiple unspecified | 28 | 0·7 | 1·4 |

### DIAGNOSIS OF BONE METASTASES

The problem of the diagnosis of a bone metastasis in a case of malignant disease is usually straightforward in that a patient with known disease, treated or untreated, presents with pain in a particular region which is X-rayed to demonstrate a change in the bone compatible with a metastasis.

The most typical X-ray finding in this series was that of decreased density (osteoporosis, radio-translucency) at the site of a metastasis or metastases. This was easily the commonest finding in the X-rays actually inspected by the author (approximately 90%), but a great number of X-rays were not actually seen (having been destroyed after a given period or having been taken in other hospitals). Many of these X-rays were not accurately reported on as to the radiological characteristics of the metastases seen, merely stating " typical secondary in such and such a bone " so that it is impossible to provide an accurate figure for the proportion of metastases showing osteoporosis.

A less typical finding is of increased bone density (osteosclerosis, radio-opacity). This was found more frequently in cases of prostatic carcinoma and also as a result of hormone treatment of breast carcinoma. Mixed osteoporotic and osteosclerotic lesions were rare. Also rare were lesions which, radiologically either osteoporotic or osteosclerotic, caused expansion of the bone concerned with the formation of a palpable lump.

### *Unknown primary*

Difficulty in diagnosis arises when a bone lesion is detected radiologically but no primary is known. In 46 instances (2·3 %) a patient first presented with a lesion which was fairly obviously a bone metastasis by clinical and radiological means, but without any easily discovered primary lesion or symptoms of such a lesion. These comprised 7·5 % of cases staged as IV. In 21 of these the primary was fairly easily detected by special investigation, but it must be emphasised that it was silent clinically. Ten of this category underwent bone biopsy.

In a further 14 cases the primary was discovered at post-mortem. The primary remained unknown in a further 11 cases for the following reasons :

(*a*) No post-mortem performed—8 cases ;
(*b*) Difficulty in proper follow-up—2 cases :
(*c*) No primary discovered even after careful post-mortem—1 case.

*Bone metastases with normal X-rays*

The commonest problem in connection with bone metastases arose when a patient with known malignant disease complained of pain compatible with metastasis but with no X-ray changes. With this situation the pain should be regarded as due to bone metastasis if it is slight at first, persists in spite of simple remedies (mild analgesics, heat treatment), increases in intensity but remains localised until ultimately it is agonising in nature.

In this series 189 cases (9·4 % of the total) had normal X-rays when first seen. This excludes patients with X-ray changes of metastases in one situation and who later developed pain in another region with normal X-rays. Once a patient is known to have metastasis, further bone pain is invariably due to further bone metastasis and a normal X-ray can be ignored. Where pain was complained of in more than one situation and X-rays were normal the site of greater pain was taken as the site of metastasis. It was found that certain sites of bone metastasis (notably vertebra) showed a high incidence of negative X-ray at the first investigation (Table V).

TABLE V.—*Cases with No Radiological or Clinical Evidence of Metastases at Onset of Pain Due to first Bone Metastasis*

| Site metastasis | Number of cases | First X-ray normal | Percentage of metastases at site |
|---|---|---|---|
| Vertebra | 1046 | 137 | 10·0 |
| Pelvis and sacrum | 357 | 29 | 3·5 |
| Femur | 127 | 5 | 1·0 |
| Ribs | 157 | 5 | 1·0 |
| Scapula | 20 | 3 | 2·6 |
| Clavicle | 26 | 2 | 2·5 |
| Sternum | 41 | 2 | 1·9 |
| Humerus | 54 | 2 | 1·0 |
| Skull | 96 | 2 | 0·7 |
| Foot | 6 | 1 | 7·7 |
| Tibia | 25 | 1 | 2·1 |

This relatively common clinical problem can be solved in the following ways :

1. Frequent repeated X-ray examination until a metastasis is demonstrated This is often unfair on the patient who may be in severe pain.

2. Specific therapy is prescribed, e.g. androgens in the instance of a breast primary, radiotherapy in most other instances. The latter method of treatment may fail if the pain cannot be accurately localised or is referred.

3. Magnification view X-rays may be of value if the appropriate apparatus is available.

4. An exploratory operation is undertaken.

5. A drill biopsy can be performed. This method was not used in this series. The difficulty is in being certain that the metastasis, if present, is actually biopsied. A negative result is thus of no significance.

### ASSOCIATED BONE PATHOLOGY

Another common problem is the patient, usually old, who presents with pain some time after treatment, usually successful, for malignant disease and in whom X-rays reveal a bone condition which could be the cause of the pain.

### *Senile osteoporosis*

Many patients with malignant disease are old or during the course of follow-up reach an age generally considered as " old ". There is, of course, no definition of old age but it is true that the older a patient becomes the greater the possibility of backache or a fracture being due to senile osteoporosis. Although occasional cases of apparent senile osteoporosis are seen in the fifties, generally speaking the diagnosis would not be considered seriously in a patient under the age of sixty. It should be noted, however, that bone metastases, particularly in the vertebrae, can closely mimic the appearance usually associated with senile osteoporosis, namely a diffuse decalcification or loss of radiological density of bone.

In retrospect it becomes easy to make a diagnosis of senile osteoporosis by virtue of the fact that the individual patient survived for a long time. On presentation, diagnosis may be supremely difficult and in fact quite frequently the patient is treated with some specific method on the assumption that bone metastases are present. Usually radiotherapy is given, often with relief of pain, when in retrospect the condition was clearly due to senile osteoporosis. However, if the patient survived for more than a year without more definite manifestations of metastatic disease the case was excluded from the series.

An instance encountered in this investigation will be considered :

*Case 6740.* Female, aged 72 : colectomy for carcinoma of splenic flexure in November 1947. Pain in the back occurred after minor trauma in July 1953 and X-rays showed widespread decalcification and collapse of D3 and D12. She died in June 1955 of a further primary in the descending colon, but with no further backache, which was thus regarded as due to osteoporosis.

### *Osteoarthritis*

This condition causes difficulty when it involves the vertebral column and there is seldom any trouble in reaching a diagnosis when other joints are affected. For example, osteoarthritis of the knee joint is common and responds as well as to be expected to simple physiotherapeutic measures.

Osteoarthritis of the spine or an apparently acutely prolapsed lumbar intervertebral disc often co-exists with metastatic malignant disease not at first visible radiologically, and only the lack of response to physiotherapeutic treatment together with eventual radiological changes can establish the diagnosis. Alternatively, response to specific therapy after a fair trial of simpler methods, if followed by the expected downhill course of the patient, can be regarded as proof that bone metastases are present.

### *Paget's disease*

This often co-exists with bone metastases. A further complication is the fact that Paget's disease of bone itself is liable to undergo malignant change. Paget's disease usually shows a typical radiological appearance, namely osteosclerosis of a large part of the affected bone, sometimes with enlargement of the bone. It is also often associated with a markedly raised serum alkaline phosphatase, an unusual finding with metastatic bone disease unless extremely widespread.

*Post-irradiation bone necrosis*

This condition must be suspected when pain persists in a part which has been irradiated. During the earlier years of the period under survey a few instances of necrosis of rib (8 cases) and of the humeral head (3 cases) following radiotherapy for carcinoma of the breast were encountered, but there were no examples in latter years as more refined radiotherapeutic techniques were used. The passage of time ultimately excludes a diagnosis of bone metastasis, as radiologically it is usually impossible to differentiate the two conditions although, on occasion, the appearances are such as to suggest the diagnosis of metastasis, e.g. if the bone lesion is sclerotic on X-ray.

Radionecrosis of the neck of the femur seems to be a common complication in some centres (Stephenson and Cohen, 1956; Koschitz-Kosic, 1961; Bickel, Childs and Porretta, 1961) but no examples were encountered in this investigation, neither were cases seen in patients undergoing pelvic irradiation and not developing bone metastases (Lederman, 1963, personal communication).

When a patient complains of pain in the hip after pelvic irradiation, particularly for gynaecologic neoplasms, radionecrosis should be suspected, as metastasis to the neck of the femur is rare with these lesions. Further radiotherapy is strongly contraindicated and the best treatment is by a prophylactic internal fixation of the femoral neck or by internal fixation if a fracture has already occurred. If there is serious doubt regarding diagnosis bone biopsy can be performed at the same time.

### PRESENTING FEATURES OF BONE METASTASES

In the series of 2,001 patients with bone metastases there was a total of 4,105 separate bony metastases. The presenting features at first attendance with bone metastasis are shown in Table VI.

TABLE VI.—*Presenting Features of* 2001 *Cases of Bone Metastasis*

| First evidence | Number of cases | Percentage of total cases |
|---|---|---|
| Pain | 1355 | 67·7 |
| Swelling | 97 | 4·9 |
| Pathological fracture | 105 | 5·2 |
| Paraplegia | 36 | 1·8 |
| Other | 12 | 0·6 |
| First detected at P.M. or fact appears on death certificate | 228 | 11·4 |
| No signs or symptoms | 425 | 21·3 |
| Unknown | 27 | 1·3 |

Swelling was noted relatively commonly with skull, sternal, tibial, mandibular, and less so with long bone metastases. Skull metastases caused a fair number of neurological symptoms and signs (relatively large in " other " column). Some patients were first seen with more than one of the features tabulated.

### TREATMENT OF BONE METASTASES

A variety of treatments have been used for the pain or other manifestations of bone metastasis in this series and these were categorised under the following headings for the purpose of this survey :

Surgery,
Irradiation,
Hormones,
Chemotherapy,
Endocrine surgery,
Others.

Treatment by irradiation will be discussed at this point and the results of various forms of hormone and hormone deprivation treatment in relationship to carcinoma of the breast and prostate will be considered later. It is fruitless to consider the various combinations of treatment possible, as the fact that a lesion did not respond to radiotherapy meant that other forms of treatment were not often tried except with breast or prostatic primaries.

In fact the results of radiotherapy generally speaking were reasonably satisfactory in the relief of symptoms of bone metastases, worthwhile relief, particularly from pain, being obtained in 357 (62 %) of 576 metastases where this method of treatment was applied (excluding cases of breast and prostatic cancer). Table VII details the findings for the major primary sites.

TABLE VII.—*Results of Radiotherapy in the Treatment of Bone Metastases*

| Primary site | Number of metastases treated | Worthwhile relief of symptoms | No improvement (including no record) | Percentage of worthwhile relief |
|---|---|---|---|---|
| Bronchus | 246 | 146 | 100 | 59·3 |
| Reticuloses | 89 | 62 | 27 | 69·7 |
| Bladder | 69 | 38 | 31 | 55·1 |
| Prostate | 35 | 21 | 14 | 60·0 |
| Kidney | 24 | 18 | 6 | 75·0 |
| Rectum | 22 | 17 | 5 | 77·3 |
| Cervix | 19 | 13 | 6 | 68·4 |
| Testis | 14 | 10 | 4 | 71·4 |
| Thyroid | 14 | 10 | 4 | 71·4 |
| Body uterus | 6 | 4 | 2 | 66·7 |
| Nasopharynx | 6 | 4 | 2 | 66·7 |
| Melanoma | 5 | 2 | 3 | 40·0 |
| Ovary | 4 | 1 | 3 | 25·0 |
| Stomach | 2 | 2 | 0 | 100·0 |
| Other (excluding breast but including unknown primary) | 56 | 30 | 26 | 53·6 |

With breast and prostatic primaries more elaborate methods of treatment were often attempted, in particular methods of hormone treatment or endocrine deprivation. In addition, multiple methods of treatment were often used and these defy analysis. However, for the 650 breast cases whose metastases (usually multiple) were treated out of 893, the methods of treatment shown in Table VIII were applied. Several methods were often applied in sequence, sometimes after response to a particular method had worn off, sometimes when one or more methods of treatment had failed.

TABLE VIII.—*Treatment of* 650 *Patients with Bone Metastases from Carcinoma Breast*

| Method of treatment | Number of cases | Worthwhile response to treatment | Percentage worthwhile response |
|---|---|---|---|
| Androgens | 343 | 147 | 42·9 |
| Oestrogens | 125 | 32 | 25·6 |
| Radiotherapy | 391 | 309 | 79·0 |
| Adrenalectomy (usually with oophorectomy) | 153 | 84 | 54·9 |
| Oophorectomy (alone) | 61 | 32 | 52·5 |
| Cortisone or similar compounds (alone) | 23 | 12 | 52·2 |
| Hypophysectomy | 66 | 19 | 28·8 |
| Chemotherapy | 29 | 5 | 17·2 |

It is seen that radiotherapy is easily the best method of treatment for the isolated metastasis. The results for adrenalectomy are somewhat better than usually reported, presumably because of the selection involved : bone metastases are well known to respond to adrenalectomy better than do soft tissue lesions. Similarly the results of hypophysectomy are worse than generally reported as almost all cases were treated by a pituitary implant of radio-active material and not by surgical extirpation. The chemotherapy group is an heterogenous collection in which various chemical compounds were tried out with very little success.

### PATHOLOGICAL FRACTURES

Paraplegia due to metastasis will be dealt with separately but can be regarded as a variety of pathological fracture. Collapse of a vertebral body not causing paraplegia was not included under the heading of pathological fracture.

### *Criteria for the diagnosis of a pathological fracture*

In most instances reasonable certainty exists that a given fracture is pathological. Occasionally difficulty is experienced in reaching such a conclusion and a firm decision may be impossible at first. This is of importance in determining treatment.

It may appear obvious that a patient should have a known primary tumour before the diagnosis of pathological fracture can be entertained. However, a certain number of patients present with a pathological fracture as the first evidence of malignant disease and in these circumstances perusal of the radiographs of the region of the fracture often reveals no definite evidence of bone metastasis.

The presence of other metastases is extremely suggestive that the fracture, particularly if sustained with a minimum of trauma, is pathological. But non-pathological fractures do occur in patients with malignant disease, particularly if old, and often with minimal trauma. For instance, several Colles fractures were known to have occurred in patients in this series, but none proved pathological. A non-pathological adduction fracture of the neck of the femur of one of these patients was treated by a nailing operation by the author, the patient, aged 82, surviving four years before dying of metastatic breast cancer not involving bone (Case 24534).

Other points to be regarded as suggesting that a fracture is pathological are as follows :

Death of the patient shortly after sustaining the fracture.

Malunion when treated by orthopaedic methods orthodox for the particular fracture.

Re-fracture soon after union.

In all there were 316 pathological fractures (excluding paraplegia) in this series (7·7 % of all metastases). Broken down into individual sites (Table IX) it will be seen that five sites (femur, ribs, humerus, clavicle and pelvis) made up the great majority of instances. However, certain other sites, e.g. tibia, provided interesting problems in treatment.

TABLE IX.—*Site of Pathological Fractures in* 4105 *Bone Metastases*

| Bone | Number of cases | Percentage of all pathological fractures | Percentage of bone metastases at site |
|---|---|---|---|
| Femur | 119 | 37·7 | 23·6 |
| Ribs | 79 | 25·0 | 15·8 |
| Humerus | 46 | 14·6 | 24·1 |
| Clavicle | 26 | 8·2 | 32·1 |
| Pelvis | 22 | 7·0 | 2·7 |
| Sternum | 8 | 2·5 | 7·5 |
| Tibia | 7 | 2·2 | 14·9 |
| Scapula | 4 | 1·3 | 3·5 |
| Radius | 3 | 0·9 | 42·9 |
| Ulna | 1 | 0·3 | 25·0 |
| Fibula | 1 | 0·3 | 11·1 |

*Pathological rib fractures*

In Table IX it will be seen that there were 79 pathological rib fractures (15·8 % of rib metastases). However, on inspecting many of the radiographs of these cases and also radiographs of rib metastases it became obvious that it was impossible to differentiate between an osteolytic lesion and a pathological fracture. It must be admitted that the proportion of pathological rib fractures depends largely on interpretation of radiographs in the absence of marked displacement of the fragments, a rare finding.

### TREATMENT OF PATHOLOGICAL FRACTURES

There is little doubt that pathological fractures of long bones due to metastases are best treated by internal fixation followed by radiotherapy (Bremner and Jelliffe, 1958). Such treatment is essential if the patient with a fractured femur is to become ambulant, although treatment with a sling is adequate to control pain due to a fractured humerus.

Table X gives details of 26 fractures treated with internal fixation. There were no examples in this series of prophylactic fixation as described by Bremner and Jelliffe.

*Pathological fractures of the femur*

Of 119, six were bilateral. In 60 the upper quarter of the bone was involved, in 51 the shaft, and in 8 the site was unknown, the fact that the femur fractured before death being noted in the death certificate or a follow-up letter. The great

TABLE X.—*Pathological Fractures Treated with Internal Fixation*

| Age at time of operation | Sex | Primary site | Site of fracture | Method of treatment | Survival in months |
|---|---|---|---|---|---|
| 59 | ♀ | Breast | Femur, upper ¼ | Nail | 7 |
| 47 | ,, | ,, | ,, ,, | ,, | 5 |
| 73 | ,, | ,, | ,, ,, | Nail-plate | 24 |
| 48 | ,, | ,, | ,, ,, | Nail | 0 |
| 54 | ,, | ,, | ,, ,, | Nail-plate | 11 |
| 57 | ♂ | Bronchus | ,, ,, | ,, ,,* | 2 |
| 45 | ♀ | Breast | ,, ,, | ,, ,, | 3 |
| 26 | ,, | ,, | ,, ,, | ,, ,, | 26 |
| 44 | ,, | Sarcomatosis–origin unknown | Rt. Femur, upper ¼ | Nail* | 3 |
| | | | Lt. ,, ,, | Nail-plate* | 2 |
| 37 | ,, | Breast | Femur, upper ¼ | Osteotomy and plate fixation | 21 |
| 52 | ,, | ,, | ,, ,, | Nail-plate | 1 |
| 52 | ,, | ,, | ,, ,, | Nail | 96 |
| 72 | ,, | ,, | ,, ,, | ,, | 4 |
| 69 | ,, | ,, | Femur, shaft | Intramedullary nail | 17 |
| 68 | ,, | ,, | ,, ,, | ,, ,, | 17 |
| 43 | ,, | ,, | ,, ,, | ,, ,,* | 6 |
| 78 | ,, | ,, | ,, ,, | ,, ,, | 4 |
| 56 | ♂ | Multiple myeloma | ,, ,, | ,, ,,* | 14 |
| 65 | ,, | Prostate | ,, ,, | ,, ,, | 9 |
| 68 | ♀ | Breast | ,, ,, | ,, ,,* | 11 |
| 57 | ,, | ,, | ,, ,, | ,, ,, | 10 |
| 53 | ,, | ,, | ,, ,, | ,, ,, | 9 |
| 77 | ,, | ,, | Tibia, shaft | Plate | 9 |
| 66 | ,, | ,, | ,, ,, | ,, | 9 |
| 66 | ♂ | Bladder | Humerus, shaft | Intramedullary Nail* | 6 |

* Operations performed by author.

majority, 78 (65·5 %), were patients with carcinoma of the breast, while carcinoma of the bronchus provided 10 cases (8·4 %). There were 5 patients with carcinoma of the bladder, 4 with multiple myelomatosis and 3 each with carcinoma of thyroid and kidney.

Twenty-three fractures (19·3 %) (one bilateral) were treated with internal fixation (9 shaft, 14 upper quarter) and these survived an average of 13·5 months after fracture. Fractures not treated with internal fixation numbered 91 (76·5 %) and of these only 5 (4·2 %) united, but 3 re-fractured before the patient's death. The average survival for this second group was 4·7 months, but there is some selection here as patients with fewer metastases and in a better general condition would be treated operatively. However, this figure of nearly 5 months represents a great deal of intensive nursing care, whereas the patient undergoing internal fixation can usually become ambulant a week or two after operation.

Two abduction fractures of the neck of the femur, both with breast primaries, survived 22 and 15 months without internal fixation.

*Pathological fractures of the humerus*

Of 46, four were bilateral. Twenty-seven (58·7 %) were due to carcinoma of the breast with 4 (8·7%) following carcinoma of the bronchus. Eleven showed other causes.

Only one shaft fracture was treated with internal fixation (bladder primary—survived 6 months after fracture). The average duration of life for the other cases was 8·4 months, which is rather longer than fractures of the femur not treated with internal fixation. There were 21 cases of shaft fracture and 19 fractures of the neck. Two fractures were supracondylar and in 4 cases the site was unknown. Three fractures (6·5 %) united, 2 breast primaries and one malignant melanoma. All had radiotherapy and one of the breast cases received hormone treatment as well. One re-fractured.

### PARAPLEGIA

In all there were 136 cases of paraplegia (6·8 % of cases with bone metastases). This amounts to 0·6 % of all cases of malignant disease (excluding rodent ulcer) during the period under review. In the series of 2,001 patients with bone metastases there were 1,376 with vertebral deposits. Thus 9·9 % of patients with vertebral metastases ultimately develop paraplegia. Nine actually developed paraplegia while being treated for vertebral metastases.

Of the patients with paraplegia, bone metastases were seen on X-ray or at post-mortem in 102 (75·0 %) while X-rays were negative and the paraplegia was assumed to be due to cord or meningeal deposits in 21 (15·4 %). In 13 (9·6 %) no X-ray or post-mortem was carried out.

Paraplegia is a complication with a very poor prognosis. In only 29 cases did the patient survive more than 6 months after the onset of the condition and of these, in 10 the paraplegia worsened while the patient underwent treatment, and 4 had no treatment so that a worthwhile and reasonably lasting response to treatment was attained in only 15 patients of 81 treated (18·5%). For the purpose of this survey these 15 patients were assigned to the category " improved ".

Two patients (2·5 %) in particular improved sufficiently to be classified as normal after treatment :

*Case 15217.*—A female aged 46 noticed a lump in the right breast in 1950. In August 1951 she developed backache but only consulted a doctor in November when she developed paraplegia. At the time the whole of the breast was occupied by a fixed carcinoma with large axillary and supraclavicular lymph nodes. The primary tumour and the spine were treated with radiotherapy. In 2 months she was walking with the aid of a walking machine and in 4 months she could walk quite normally. She survived for 17 months.

*Case 8457.*—A male aged 40 developed paraplegia in June 1945. At laminectomy a multiple myeloma deposit was removed with complete recovery of the paraplegia. In the following 4 years several episodes of pain in the back were treated with radiotherapy, with relief, before he died of widespread disease.

On the other hand one patient survived with unimproved paraplegia for as long as 48 months :

*Case 7588.*—A male aged 35 had a right orchidectomy carried out in 1948 for a seminoma. In 1950 the left testicle was removed for a similar tumour. In 1951 he developed paraplegia and an extradural metastasis was removed and radiotherapy given. He died, unimproved in 1955.

Table XI shows the primary sites for the patients with paraplegia.

TABLE XI.—*Primary Disease in 136 Patients with Paraplegia*

| Primary site | Number of cases | Percentage of cases with paraplegia | Percentage of cases at the primary site with bone metastases |
|---|---|---|---|
| Breast | 47 | 34·6 | 4·7 |
| Bronchus | 22 | 16·2 | 6·5 |
| Reticulosis | 17 | 12·5 | 18·3 |
| Prostate | 8 | 5·9 | 7·5 |
| Testis | 7 | 5·1 | 36·8 |
| Kidney | 5 | 3·7 | 16·1 |
| Bladder | 5 | 3·7 | 4·3 |
| Other | 25 | 18·4 | — |

*Treatment*

The average duration of life with paraplegia was 4·3 months. For the treated group the figure was 7·2 months.

Fifty-five patients received no treatment for their paraplegia. The average duration of survival for this group was 2·0 months. It must not be concluded that treatment would have lengthened survival: many of these patients were untreated because of their poor general condition, e.g. only 8 of 22 patients with carcinoma of the bronchus were treated.

Multiple methods of treatment were used in a number of patients and with these it was usually found possible to decide which of consecutive treatments afforded worthwhile palliation for a reasonable length of time.

Treatment could be divided into four categories:

(i) *Radiotherapy*

| | |
|---|---|
| Number of cases treated | 56 |
| Paraplegia improved but survived less than 6/12 | 4 |
| Paraplegia improved and survived more than 6/12 | 9 |
| No improvement | 43 |

(ii) *Laminectomy*

| | |
|---|---|
| Number of cases treated | 15 |
| Improved | 3* |
| No improvement | 12 |

(iii) *Hormone therapy* (including endocrine deprivation surgery) which was used on 17 cases of carcinoma of breast and 5 of carcinoma of prostate. The latter group is too small to consider in detail except to state that three patients survived improved for 51, 41 and 19 months with oestrogens, together with R.T. (2nd of these cases) and laminectomy (3rd). Of the 17 breast cases treated 3 improved with androgens (31, 19 and 17 months) and 6 received no worthwhile benefit. The other cases were treated with adrenalectomy (4 patients), hypophysectomy (destruction with radioactive sources; 3 patients), and cortisone (1 patient) with no benefit.

(iv) *Chemotherapy*: (Miscellaneous). Three patients were treated; one with a testicular primary improved (7 months).

* Three patients showed improvement with more or less simultaneous therapy in which the improvement could not definitely be assigned to the laminectomy:

(1) Carcinoma prostate: Laminectomy + oestrogens (19 months).
(2) Multiple myeloma: Laminectomy + radiotherapy (48 months).
(3) Neuroblastoma: Laminectomy + radiotherapy (9 months).

The patients with paraplegia due to metastasis from carcinoma of the breast were sufficiently numerous to warrant a more detailed analysis. Nineteen untreated cases were mostly seen early in the period of review and survived an average of 2·8 months. Twenty-eight treated cases survived an average of 7·5 months. It is likely that in this instance the difference in period of survival is not due wholly to selection of patients in better condition.

Paraplegia due to carcinoma of the breast is relatively common and from the results of treatment obtained in this series the conclusion is reached that it must be energetically treated by radiotherapy together with androgens, preferably at the stage when the patient complains of pain in the back, and before the condition actually develops.

### SUMMARY

A series of two thousand patients with secondary malignant disease of bone is reviewed. Patients with carcinoma of the breast comprised half of these. A quarter of sufferers from carcinoma of the breast ultimately will develop bone metastases.

The traditional text book sites of origin of bone metastases (breast, prostate, kidney, thyroid, bronchus) were confirmed.

The chances for a patient with bone metastasis of involvement of the vertebrae, pelvis and femora are respectively 70, 40 and 25 per cent.

When first seen with bone metastases nine per cent of patients have normal X-rays. For vertebral metastases this figure rises to thirteen per cent.

Radiotherapy is easily the best method of relieving the pain of bone metastasis. Seventy per cent of all patients are appreciably benefited.

A quarter of the metastases involving the long bones will cause pathological fractures and of these only 5 per cent will unite without internal fixation. This method of treatment, if applied early for the patient who has more than a week or two to live, will save much nursing care and alleviate a great deal of pain. In half the patients so treated a long period of pain-free use of the limb is attained, before disseminated disease causes death.

Of the patients with paraplegia due to metastasis, carcinoma of the breast is the cause in a third. They should be treated energetically with androgens combined with radiotherapy. There is little evidence that laminectomy improves the results of treatment of paraplegia due to metastasis.

I wish to thank the British Empire Cancer Campaign for Research for a grant during the tenure of which this work was carried out, and the Clinicians of the Royal Marsden Hospital for allowing me to use their case material. Dr. N. F. C. Gowing has kindly reviewed numerous histological diagnoses. Mr. P. M. Payne of the South Metropolitan Cancer Registry had given valuable statistical help and Miss S. Taylor has helped similarly with follow-up records.

### REFERENCES

Bickel, W. H., Childs, O. S. and Porretta, C. M. (1961) *J. Amer. med. Ass.*, **175**, 204.
Bremner, R. A. and Jelliffe, A. M. (1958) *J. Bone Jt. Surg.*, **40**B, 652.
Koschitz-Kosic, H. (1961) *Klin. Med.*, **16**, 277.
Stephenson, W. H. and Cohen, B. (1956) *J. Bone Jt. Surg.*, **38**B, 830.